# Quantitative Imaging of Bacteriophage Amplification for Rapid Detection of Bacteria in Model Foods

**DOI:** 10.3389/fmicb.2022.853048

**Published:** 2022-03-04

**Authors:** Nicharee Wisuthiphaet, Xu Yang, Glenn M. Young, Nitin Nitin

**Affiliations:** ^1^Department of Food Science and Technology, University of California, Davis, Davis, CA, United States; ^2^Nutrition and Food Science Department, California State Polytechnic University Pomona, Pomona, CA, United States; ^3^Department of Biological and Agricultural Engineering, University of California, Davis, Davis, CA, United States

**Keywords:** bacteriophage amplification, *Escherichia coli*, pathogen detection, fluorescence imaging, qPCR

## Abstract

Rapid detection of bacteria in water and food samples is a critical need. The current molecular methods like real-time PCR can provide rapid detection after initial enrichment. However, these methods require significant preparation steps, specialized facilities to reduce contamination, and relatively expensive reagents. This study evaluates a novel approach for detecting bacteria based on imaging of bacteriophage amplification upon infection of the target host bacteria to mitigate some of these constraints and improve the specificity of discriminating live vs. dead bacteria. Thus, this research leverages the natural ability of lytic bacteriophages to rapidly amplify their genetic material and generate progeny phages upon infecting the host bacterium. This study uses a nucleic acid staining dye, a conventional fluorescence microscope, and quantitative image analysis for imaging the amplification of bacteriophages. The sensitivity and assay time for imaging-based quantification of phage amplification for detecting *Escherichia coli* were compared with RT-PCR and the standard plaque-forming assay for detection phage amplification in model systems, including coconut water and spinach wash water. The results demonstrate that the imaging approach matches both the sensitivity and speed for detecting *E. coli* using the RT-PCR method without requiring isolation of nucleic acids, expensive reagents, and specialized facilities. The quantitative imaging results demonstrate the detection of 10 CFU/ml of *E. coli* in coconut water and simulated spinach wash water with a chemical oxygen demand (COD) of 3,000 ppm within 8 h, including initial enrichment of the bacteria. In summary, the results of this study illustrate a novel phage amplification-based approach for detecting target bacteria in complex food and water samples using simple sample preparation methods and low-cost reagents.

## Introduction

Detection of pathogens and indicator bacteria in food and water systems is one of the essential tools to manage the risk of foodborne outbreaks. Common pathogenic bacteria, including *Escherichia coli*, *Listeria monocytogenes*, and *Salmonella* spp., have been reported as the major causes of bacterial foodborne illness outbreaks (Bintsis, 2017). Current methods of detecting these bacteria include the traditional plate counting assay or more rapid molecular methods such as PCR or ELISA (Gracias and McKillip, 2004). Among diverse bacteria associated with foodborne illnesses, *Escherichia coli* are a Gram-negative bacterium typically found in the enteric tract of humans and warm-blooded animals (Kaper et al., 2004). Even though most *E. coli* strains are not pathogenic, *E. coli* presence is an indicator for fecal contamination and, therefore, their presence indicates the potential contamination of water and food supplies by other pathogenic bacteria (Krumperman, 1983; Molina et al., 2015; Yang et al., 2017). Thus, *E. coli* is one of the well-established indicator microorganisms for ensuring the hygiene and safety of food products (Choi et al., 2018). In addition, there are specific strains of *E. coli* that are pathogenic to humans, including enteropathogenic *E. coli* (EPEC), enterotoxigenic *E. coli* (ETEC), enteroinvasive *E. coli* (EIEC), enteroaggregative *E. coli* (EAEC), and enterohemorrhagic *E. coli* (EHEC; Yang et al., 2017). Thus, improving detection of *E. coli* can enable monitoring of general sanitation and hygiene in food environments and enable detection of specific pathogens in food and water systems.

For detecting diverse pathogenic and indicator bacteria in food and water systems, culture-based methods with selective media are the most established approaches used routinely by diverse sections of the food and agriculture industries (De Boer and Beumer, 1999; Feng et al., 2020). However, this detection approach is labor-intensive and often requires several days to obtain the results (Wang and Salazar, 2016). Complementary to the culture-based assays, molecular assays based on nucleic acid-based amplification and detection, such as PCR, are commonly used for the detection of both indicator and pathogenic bacteria, including *E. coli* (Molina et al., 2015; Choi et al., 2018; Feng et al., 2020). Even though PCR offers rapid and specific detection, it has limited sensitivity to differentiate between viable and non-viable cells (Yuan et al., 2018). Among immunological-based rapid detection approaches, ELISA is one of the most widely used methods for bacterial detection. ELISA utilizes the specific antigen-antibody interaction and the catalytic properties of an enzyme to provide a sensitive detection (Gracias and McKillip, 2004; Pang et al., 2018). ELISA can be automated and analyzed a large sample number simultaneously (Ivnitski et al., 1999). For example, Vidas (bioMérieux) is a fully automated system developed to detect bacteria such as *Listeria monocytogenes* using an enzyme-linked fluorescent assay technology (Gangar et al., 2000). However, like PCR, it cannot distinguish between live and dead cells (De Boer and Beumer, 1999). Detecting live/viable bacteria is of interest to food producers to eliminate false positives due to residual dead microbes or molecules of microbial origin left behind following food processing and sanitation. In addition, these aforementioned rapid detection approaches share some drawbacks such as limited sensitivity when the samples contain a low number of target bacteria, interference from the food components and significant sample preparation steps in the case of PCR methods, and limited stability of immunoreagents for immunoassays (Jaykus, 2003; Hornbeck, 2015). In addition, many molecular approaches such as PCR methods also rely on the enrichment of bacteria before detection. In many cases, these enrichment processes can range from 6 to 24 h before sample preparation steps, such as isolation and purification of nucleic acids for PCR assay (Choi et al., 2018; Manage et al., 2019). Therefore, there is a need to develop molecular-specific detection methods using low cost, stable reagent, simple sample preparation steps, detection within 6–8 h of sample collection, and reduce the need for specialized laboratory environments.

Among various options, bacteriophages or phages can enable low-cost detection of target bacteria with simple sample preparation methods. For *E. coli* detection, the T7 phage is a well-established model system due to its ability to infect a wide range of *E. coli* hosts (Studier, 1969). With genetically modified T7 phages, previous studies have illustrated detection of 10–100 CFU/ml of target bacteria in water and beverage samples within 6–8 h without isolation of nucleic acids or extensive sample preparation steps (Chen et al., 2015, 2017; Wisuthiphaet et al., 2019, 2021). However, the application of this approach requires genetic modification of phages. It limits the broad applicability of phage-based detection methods, especially given the enormous diversity of phages in the environment. In addition, these genetically modified phage assays require isolation of the enzyme after its expression in target bacteria to increase the sensitivity of detection (Hinkley et al., 2018; Tilton et al., 2019). Furthermore, in certain instances, background levels of enzymatic activity in the non-targeted and targeted bacteria and the food products can interfere with the detection approach (Talbert et al., 2016; Meile et al., 2020).

We have evaluated the potential of morphological changes induced by phage lysis to detect bacteria for addressing some of these constraints. This approach could detect 10 CFU/ml of *E. coli* in 8 h (Yang et al., 2020). Similar to the concept of morphological changes in the bacteria, the lytic phage infection of the host cell also generates significant and rapid amplification of the progeny phage particles. For example, infection of an *E. coli* cell with a T7 phage may generate more than 100 progeny phages within 25–30 min. Thus, phage infection of target bacteria can result in significant amplification of phage particles. The presence of bacteria in media and food matrices can be detected based on the isolation of DNA from amplified phages and its subsequent amplification using a real-time qPCR technique (Tolba et al., 2008; Anany et al., 2017). Although PCR is considered a rapid and sensitive technique for measuring bacteriophage amplification assay, it requires DNA isolation and several sample-preparation steps to prolong the detection process. In addition, the presence of inhibitors from food matrices can influence nucleic acid amplification efficiency. Phage amplification can also be detected using a conventional culture-based method like plaque assay, but it requires an extended incubation time to allow the growth of bacterial lawn to enumerate phage particles (Anderson et al., 2011).

This study aims to develop an approach for phage amplification detection using fluorescence imaging and quantitative image analysis to address these limitations. After phage lysis of the target bacterial host, the amplified phages were enriched by removing the bacterial debris using a simple centrifugation step. The phage enriched solution was stained with a nucleic acid stain (SYBR green I), and the stained phage particles were enumerated based on quantitative imaging measurement. Based on this approach, this study demonstrates both visualization and quantification of the amplification of T7 phages upon infecting *E. coli*. Sensitivity and the total assay time for this novel approach were compared with the qPCR detection of phage DNA amplification and the plaque assay. The developed imaging-based detection method was then applied to detect *E. coli* in model food systems. Coconut water was selected as a sample representing beverage matrices containing sugar, lipids, and minerals (Prades et al., 2012). Simulated spinach wash water was chosen to represent a complex matrix related to fresh produce production since several *E. coli* outbreaks are related to the consumption of leafy greens. Postharvest cross-contamination is one of the key risk factors associated with these outbreaks (hydro cooling or washing; Gil et al., 2009).

The key advantages of this approach include the use of non-genetically modified phages to detect low concentration of bacteria in liquid food samples. In addition, the imaging method developed in this study for the detection of amplified phage particles following infection of the target bacteria provides an alternative approach to detect bacteria using qPCR methods for the detection of amplified phages (Stanley et al., 2007; Kutin et al., 2009; Anany et al., 2018; Garrido-Maestu et al., 2019; Malagon et al., 2020). Furthermore, the approach in this study does not require immobilization of phages on substrates or paper strips to reduce the background signal from parent phages as observed in the previous study (Anany et al., 2018).

In summary, this novel image-assisted quantitative phage amplification detection approach can provide a robust yet straightforward strategy to address some of the critical unmet needs for detecting target bacteria in food and environmental samples using low cost, stable reagents, and limited sample preparation steps.

## Materials and Methods

### Bacteriophage and Bacterial Strain

T7 phages were purchased from American type culture collection (#BAA-1025-B2) and propagated by infecting the log-phase *E. coli* BL21 for 15 min at 37°C in an incubator-shaker. Then, the phage-bacteria mixture was centrifuged at 16,100 × *g* to harvest infected bacterial cells. The cells were then resuspended in sterile tryptic soy broth (TSB, Sigma-Aldridge, St. Louis, MO, United States) and incubated at 37°C with 200 rpm constant shaking for complete lysis for 3 h or until no visible turbidity was observed. Chloroform was then added to the mixture to a final concentration of 20% (vol/vol) and vortexed vigorously for homogeneity. The mixture was incubated on ice for at least 10 min before centrifugation at 5,000 × *g* for 10 min to precipitate the cell debris. The upper phase of TSB containing phage particles was collected and washed three times by resuspending in a sterile phosphate buffer solution (PBS, Fair Lawn, NJ, United States) and centrifugation at 16,100 × *g* for 10 min. After washing, the phage particles were resuspended in PBS, and the phage titer was enumerated as 10^8^ PFU/ml. The phage stock was stored at 4°C until used.

*Escherichia coli* BL21 (ATCC BAA-1025) obtained from American type culture collection was used as the host for the T7 phages. The bacterial culture was stored in TSB containing 15% (vol/vol) glycerol at −80°C. For short-term storage, the glycerol stock of bacteria was aerobically grown overnight in TSB at 37°C with constant shaking at 200 rpm. The culture was streaked onto tryptic soy agar (Sigma-Aldridge, St. Louis, MO, United States) plates and incubated overnight at 37°C. The agar plates were stored at 4°C for further experiment.

### Amplification of T7 Using Various Concentrations of *Escherichia coli* Host Cells for Further Enumeration

A single colony of *E. coli* BL21 was inoculated in TSB and incubated aerobically at 37°C with constant shaking at 200 rpm for 16 h to obtain a 10^9^ CFU/ml concentration. The bacterial culture was washed twice and resuspended in a sterile PBS before serial dilution. Then, the *E. coli* BL21 suspension was serially diluted in 10 ml TSB to obtain solutions with 10–10^3^ CFU/ml of bacterial cell concentration. These solutions were then individually enriched by incubating at 37°C with constant shaking at 200 rpm for 4 h.

T7 phages were added to the 4-h enriched *E. coli* with constant shaking at 37°C for 0–4 h. Chloroform (20% vol/vol) was added to the mixture, followed by incubation on ice for 5 min before centrifugation at 16,100 × *g* for 10 min. The upper liquid phase containing bacteriophage particles was collected for analysis. The samples were analyzed using the standard plaque counting assay, RT-PCR, and quantitative imaging.

### Fluorescence Imaging of Bacteriophage Particles

**Figure 1:**
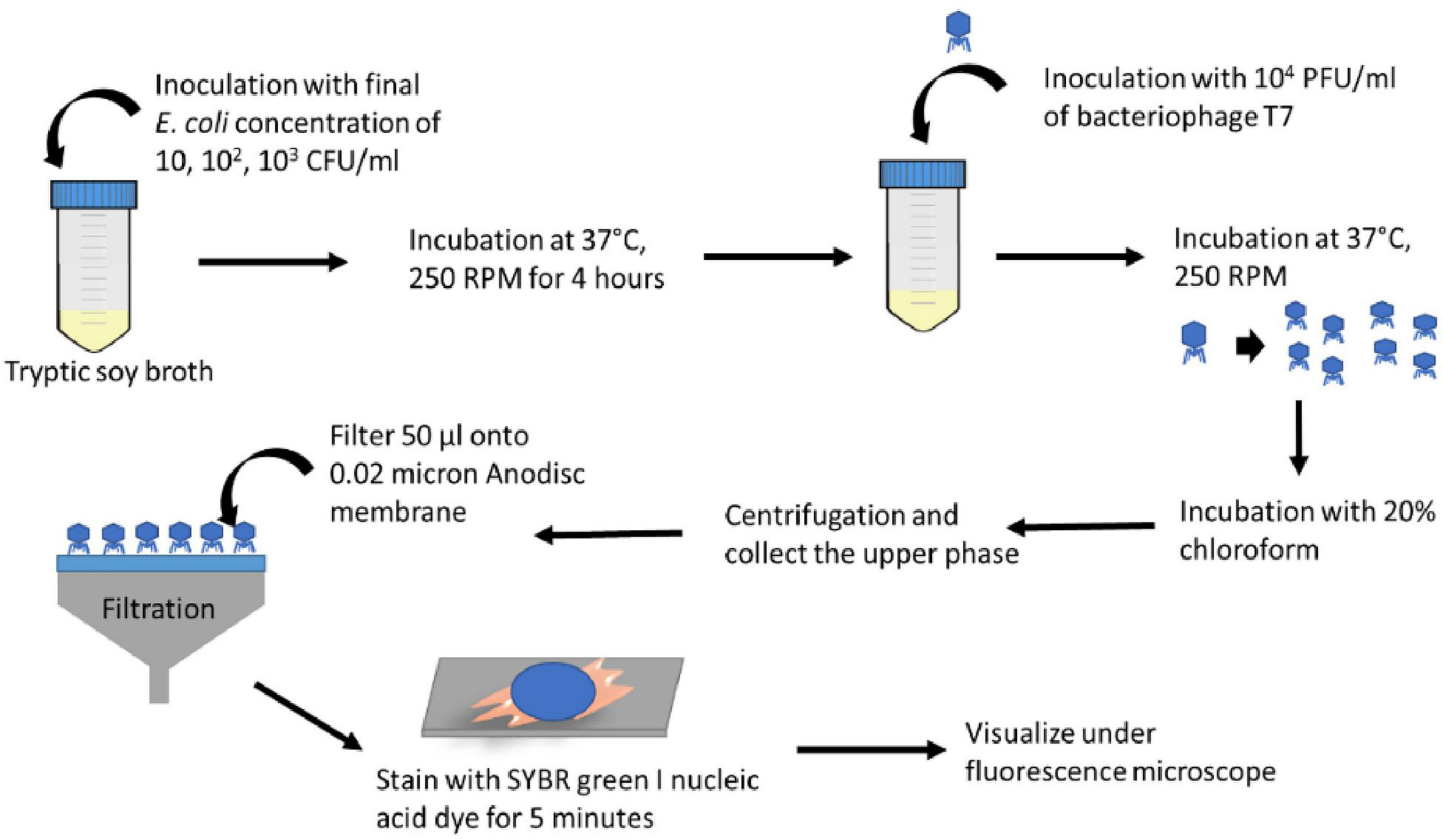
Schematic diagram illustrating the steps for the bioassay based on imaging of progeny phages after infecting target bacteria.

Figure 1 illustrates the overall procedure for detecting bacteria using a combination of phage and an imaging approach. After T7 phage amplification, 50 μl of the phage solution was filtered through a 0.02-μm pore size Whatman® Anodisc inorganic filter membrane with a 13-mm diameter (GE Healthcare, Buckinghamshire, United Kingdom) and air-dried for 1–2 min. The Anodisc filter was then put back-side-down onto 20 μl of SYBR green I (×25) spotted on the glass slide and kept in the dark for 5 min. After staining, the excess dye was removed by Kimwipe, followed by adding 20 μl of 1% of *p*-phenylenediamine on top of the filter as an antifading reagent (Patel et al., 2007). The Anodisc filter was covered with a coverslip and observed under the Olympus IX-71 inverted fluorescence microscope with a ×100 (1.25 NA) objective lens. An average of 10–12 images was acquired for each Anodisc sample using the Metamorph imaging software (version 7.7.2.0, Universal Imaging Corporation). The fluorescence excitation/emission wavelength of SYBR Green I stain was 480 ± 30 and 535 ± 40 nm, respectively.

T7 phage stock solution was serially diluted in PBS to have concentrations of 10^4^–10^8^ PFU/ml before fluorescence imaging for evaluating the limit of detection of T7 concentration by this imaging method.

### Image Analysis for Bacteriophage Amplification Enumeration

The acquired fluorescence images were analyzed using a custom MATLAB code (Section 1 in the Supplementary Information). To segment the phage particles in the fluorescence images, the *strel* function was used to define the shape of the phage particles, which were disk-shaped elements with a radius smaller than 30 pixels. Subsequently, the function *imtophat* was used to remove uneven background, followed by *medfilt2* function for reducing background noise. The function *adapthisteq* was used to enhance the contrast of the grayscale image and improve the visibility. The function *wiener2* was used to remove fine noise where each output pixel contains the median value in the 3-by-3 neighborhood around the corresponding pixel in the input image. The segmented images were then converted to binary images using the *imbinarize* function based on the intensity threshold computed by the *adaptthresh* function. For enumerating the number of particles, the function *bwlabel* was used to label bacteriophage particles in a binary image and count the number of particles present in the images as an output.

### Quantification of Bacteriophage by Real-Time qPCR

The amplified phage particles from the section “Amplification of T7 Using Various Concentrations of *Escherichia coli* Host Cells for Further Enumeration” were also quantified by real-time qPCR. The phage solution was heated at 100°C for 15 min for denaturation of the phage particles and release of the phage DNA. The real-time qPCR assay was performed using the forward (5′-CCT CTT GGG AGG AAG AGA TTT G-3′) and reverse (5′-TAC GGG TCT CGT AGG ACT TAA T-3′) primers (Peng et al., 2018) designed from partial genome sequence of T7 phage. The PCR reaction was conducted with the total volume of 10 μl containing 5 μl of PowerUp SYBR Green Master mix ×2 (Life Technologies, Grand Island, United States), 1 μl of 100 μM primers, 2 μl of UltraPure™ DNase/RNase-Free Distilled Water (Invitrogen Life Science Technologies), and 2 μl of the denatured T7 phage DNA. The thermocycler condition were 2 min at 50°C, 2 min at 95°C followed by 40 cycles of 15 s at 95°C, and 1 min at 60°C. The melt curve setting was 15 s at 95°C, 1 min at 60°C, and 15 s at 95°C. The Ct value of the samples was recorded. The concentration of the phage DNA was calculated based on the standard curve prepared by using purified T7 phage DNA (39.9 kbp, Boca Scientific) and reported in picogram units (pg).

### Plaque Assay

The plaque assay of the amplified phage solutions obtained from the section “Amplification of T7 Using Various Concentrations of *Escherichia coli* Host Cells for Further Enumeration” was performed using a soft agar prepared by supplementing the TSB broth with 0.7% agar before sterilization. A 10 ml of the molten soft agar was aliquoted to a sterile 15-ml centrifuge tube and cooled down to less than 50°C. As described in the section “Amplification of T7 Using Various Concentrations of *Escherichia coli* Host Cells for Further Enumeration,” the amplified phage solution was 10-fold serial diluted in a sterile PBS. A 100 μl of each dilution of the phage solution was mixed with the aliquoted soft agar along with 100 μl of *E. coli* BL21 overnight culture. The mixture was then poured into a sterile Petri dish (10 cm diameter). The plates were gently rocked to distribute the soft agar on the Petri dish surface homogeneously. After the soft agar was solidified, the plates were inverted and incubated overnight at 37°C. The titer of the phage particles was calculated based on the number of plaques formed on the agar and reported in PFU/ml.

### Detection of *Escherichia coli* in Coconut Water and Simulated Spinach Wash Water

Baby spinach was purchased from a local grocery store. Spinach of 50 g was combined with 500 ml sterile water before blending two times using a sanitized blender for 30 s at a maximum speed. The blended solution was then centrifuged for 10 min at 11,000 × *g*. The supernatant was collected and centrifuged again at the same speed. The supernatant was collected as the simulated spinach wash water. The spinach wash water’s chemical oxygen demand (COD) was measured to be approximately 3,000 mg/L. Coconut water (Vita coco 100% coconut water) was purchased from a local grocery store.

For bacterial enrichment, 5 ml of coconut water (Vita coco 100% coconut water) and 5 ml of simulated spinach wash water were mixed with 5 ml double concentrated TSB before inoculation with 10 CFU/ml of *E. coli* and incubation at 37°C with constant shaking at 200 rpm for 4 h. The enriched mixture was inoculated with T7 phage for infection of the target bacteria. The lysate was isolated for quantifying phage amplification using the image-based, PCR, and plaque assay methods described above.

### Statistical Methods

The phage particle number and phage DNA concentration obtained from both the imaging and qPCR measurements, respectively, at each infection time, were converted to a log scale value (*y*) and normalized by dividing by the log value of the maximum phage count assessed based on these measurements (*y*_0_). The normalized values (*y*/*y*_0_) of phage level quantified by both methods were fitted to equation 1 using the curve fitting toolbox in MATLAB. The parameter *k* (sec^−1^), indicating the rates of bacteriophage amplification quantified by the imaging and PCR methods, were obtained. The linear correlation between both imaging-plaque assay and PCR-plaque assay was obtained using OriginPro 9.1 software.


(1) $$\frac{y}{y0}=1-e^{-kt}$$

## Results and Discussion

### Identification of Initial Phage Inoculum Concentration

The overall goal of this experiment was to determine the threshold concentration of phages that can be detected using a simple imaging approach. This threshold concentration will establish the initial inoculum concentration of phages for infecting bacteria while generating minimal background signal in the imaging measurement.

**Figure 2:**
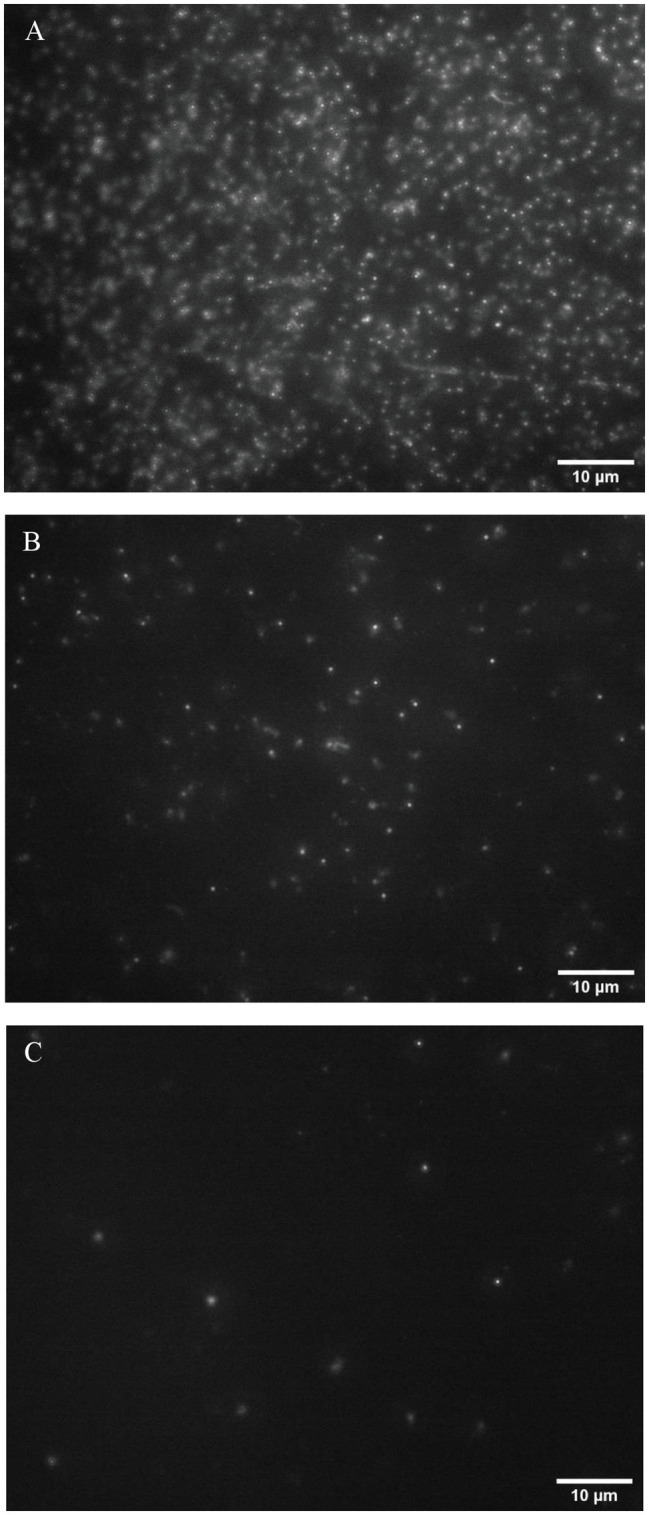
Fluorescence images of the bacteriophage particles at concentration levels of **(A)**10^8^ PFU/ml, **(B)** 10^7^ PFU/ml, and **(C)** 10^6^ PFU/ml.

Phage particles were visualized by fluorescence imaging after staining with a nucleic acid binding dye SYBR green I. The method used in this study was modified from the earlier work by Patel et al. (2007), where this fluorescence imaging was used for investigating virus-like particles from the marine environmental samples (Patel et al., 2007). By filtration, bacteriophages were captured on a 13-mm diameter Anodisc filter with 0.02-micron pore size. T7 phages with a concentration range between 10^4^ and 10^8^ PFU/ml were imaged using this approach. Figure 2 shows fluorescence images of phage particles with an initial concentration of 10^8^, 10^7^, and 10^6^ PFU/ml. The phage particles appeared as small dots homogeneously distributed on an Anodisc filter. Phages with a concentration lower than 10^5^ PFU/ml were not detectable using the imaging settings and configuration selected for this study. This measurement establishes that the initial phage inoculum levels at 10^4^ or 10^5^ PFU/ml may not generate significant background staining. The results also illustrate that amplifying the phage titer at least 10^6^ PFU/ml after infection of the target bacteria enables detection of phage particles using a conventional fluorescence microscopy. Since amplification of T7 phage results from phage infection and lysis of their bacterial host *E. coli* cells, detection of T7 amplification after phage inoculation, and incubation with samples will indicate the presence of *E. coli* contamination. T7 phage has a short infection and lysis time, and it releases more than 100 copies of its progeny phages after 25 min of the initial infection (Qimron et al., 2010), 10^4^ PFU/ml of phage concentration was selected as an initial inoculum for this study. An increase in phage concentration at or above 10^6^ PFU/ml upon infection will enable specific detection of the presence of the target bacteria.

### Detection of Target Bacteria Based on Bacteriophage Amplification and Imaging

The overall goal of this sub-task was to determine the influence of infection time on the levels of amplified phages for a range of initial bacterial concentrations and to correlate the imaging-based phage amplification measurements with the conventional plaque assay.

Before T7 phage infection, *E. coli* BL21 with an initial concentration between the range of 10 and 10^3^ CFU/ml were enriched in TSB for 4 h. This step mimics the typical enrichment process used for both the culture-based and rapid detection methods, including the qPCR methods to detect bacteria in food and water systems. After enrichment and infection with T7 phage, the samples were collected at 30-min intervals for the subsequent 4-h period. After collection, the samples were filtered, labeled, and imaged, as illustrated in Figure 1.

**Figure 3:**
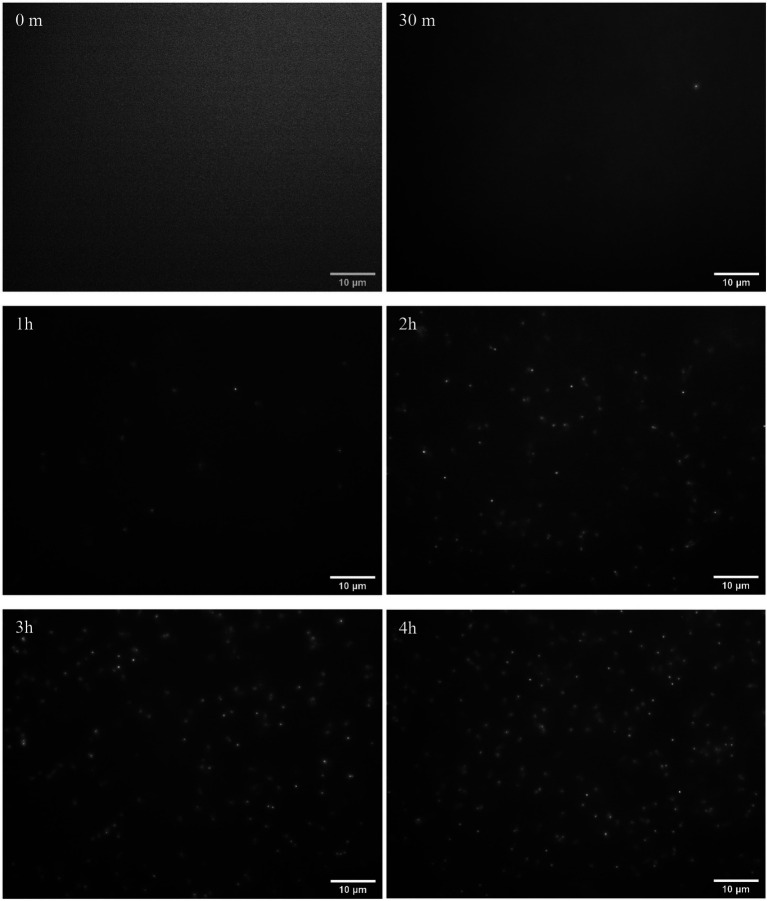
Fluorescence images of the bacteriophage particles after 0 min, 30 min, 1 h, 2 h, 3 h, and 4 h of infection of *Escherichia coli* BL21 (10^2^ CFU/ml initial concentration) with 10^4^ PFU/ml of T7 phages. *Escherichia coli* cells were enriched for 4 h prior to infection with phages.

The examples of fluorescence images of phage particles are shown in Figure 3. The images were obtained when the initial bacteria concentration was 10^2^ CFU/ml with 0 min, 30 min, 1 h, 2 h, 3 h, and 4 h of phage infection. All of the acquired images were analyzed using the image analysis approach described in the section “Image Analysis for Bacteriophage Amplification Enumeration.” The examples of images obtained from the initial *E. coli* concentration of 10 and 10^3^ CFU/ml are shown in Section 2 in the Supplementary Information.

**Figure 4:**
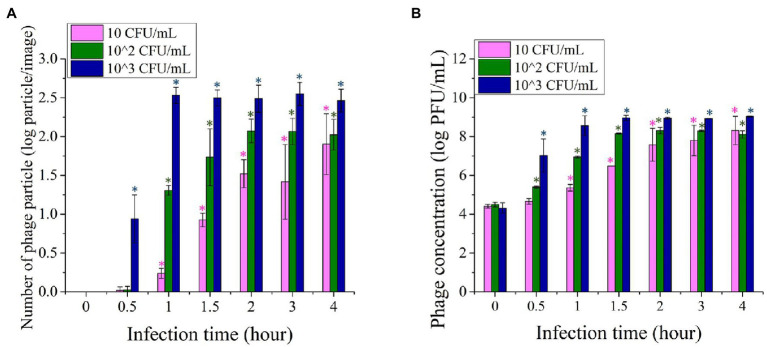
**(A)** Imaging based quantification of the increase in the number of phage particles upon infection of *Escherichia coli* BL21 as function of infection time (0–4 h). **(B)** Quantification of plaques generated based on infection of *E. coli* BL21 using the standard plaque assay. For both of these assays, *E. coli* cells with initial concentration levels of 10, 10^2^, and 10^3^ CFU/ml were enriched for 4 h prior to infection with phages. Treatments with “*” are significantly different (*p* < 0.05) from control at 0 min infection. Error bars indicate ±SD of means.

Figure 4A shows the number of phage particles enumerated based on image analysis of the fluorescence images acquired using 10, 10^2^, and 10^3^ CFU/ml of the initial concentration of bacterial inoculum. For each initial concentration level of bacteria, 10–12 images were acquired and analyzed for phage particles quantification. The initial bacteria concentration was enriched for 4 h and infected with T7 phages at 10^4^ PFU/ml. Immediately after introducing the T7 phage at 10^4^ PFU/ml to the enriched samples, no phage particles were observed in the fluorescence images as the fluorescence signal was below the detection limit. When the initial concentration of *E. coli* was 10 CFU/ml, after 4 h of enrichment and 1-h infection, fewer than 10 phage particles/image were observed, where each image represents an area of 6 × 10^3^ μm^2^. The number of phage particles increased to 30–50 particles/image after 2 h of infection, and around 100 particles/image was observed within 4-h of infection. With an increase in the initial *E. coli* concentration to 10^2^ CFU/ml, after 4-h enrichment and 30 min of infection, fewer than five bacteriophages/image particles were visualized. The number of phage particles in the images increased to around 10–20 particles/image after 1-h infection and to more than 100 phage particles/image after 2 h of infection. The number/image remained constant after 3 and 4 h of phage infection of target bacteria. For the initial *E. coli* concentration of 10^3^ CFU/ml, the number of particles increases more rapidly to around 10 phage particles/image within the first 30 min of infection and subsequently increased to 300–400 phage particles/image after a 1-h incubation. The number of phage particles remained constant with a longer infection time for 10^3^ CFU/ml initial inoculum. According to the results, *E. coli* at 10 and 10^2^ CFU/ml can be detected using the phage amplification-based imaging method after 4 h of enrichment followed by 1 h of phage infection. A 4-h enrichment and 30-min phage infection time were required to detect 10^3^ CFU/ml of *E. coli*.

The increase in the numbers of phage particles in the fluorescence images was comparable with the titer of bacteriophage enumerated by the plaque assay. Figure 4B illustrates the titer of phage obtained by the standard plaque assay after infection of a 4-h enriched *E. coli* with T7 phages at 10^4^ PFU/ml. When the initial concentration of *E. coli* was 10 CFU/ml, it required approximately 1.5 h of infection time for the phage titer to reach the level of 10^6^ PFU/ml. The concentration of 10^6^ PFU/ml was the lowest level of phage count that can be detected by fluorescence imaging, as illustrated in Figure 1. When the initial concentration of *E. coli* was 10^2^ and 10^3^ CFU/ml, 10^6^ PFU/ml of T7phages can be achieved after 1 h and 30 min of infection time, respectively.

The results also show that the amplification rate of phage depends on the concentration of bacteria. This trend was in agreement with a previous study that described the phage bacterial binding process as dependent on both the concentration of free phages and the concentration of bacteria (Ellis and Delbrück, 1939; Sinha et al., 2018). Thus, higher initial *E. coli* concentration results in a higher phage amplification rate and thus a relatively higher titer of bacteria (initial concentration of 10^3^ or 10^4^ CFU ml) after enrichment can be detected with a short infection time. In addition, due to a rapid lytic cycle of the T7 phage, a significant amplification of the phages with each lytic cycle and sensitivity of the imaging approach, even multifold lower levels of bacteria, for example, 10 CFU/ml could also be detected within 6 h. This highlights the overall advantage of this approach that enables rapid and sensitive detection of target bacteria at levels as low as 10 CFU/ml within 6 h for enrichment and infection. This result also highlights that the combination of phage amplification and sensitivity of an imaging approach can reduce the influence of multifold differences in the bacterial titer, e.g., a 100-fold difference between 10 and 10^3^ CFU/ml after enrichment.

### Comparison of Phage Quantification Using Imaging and qPCR Methods

**Figure 5:**
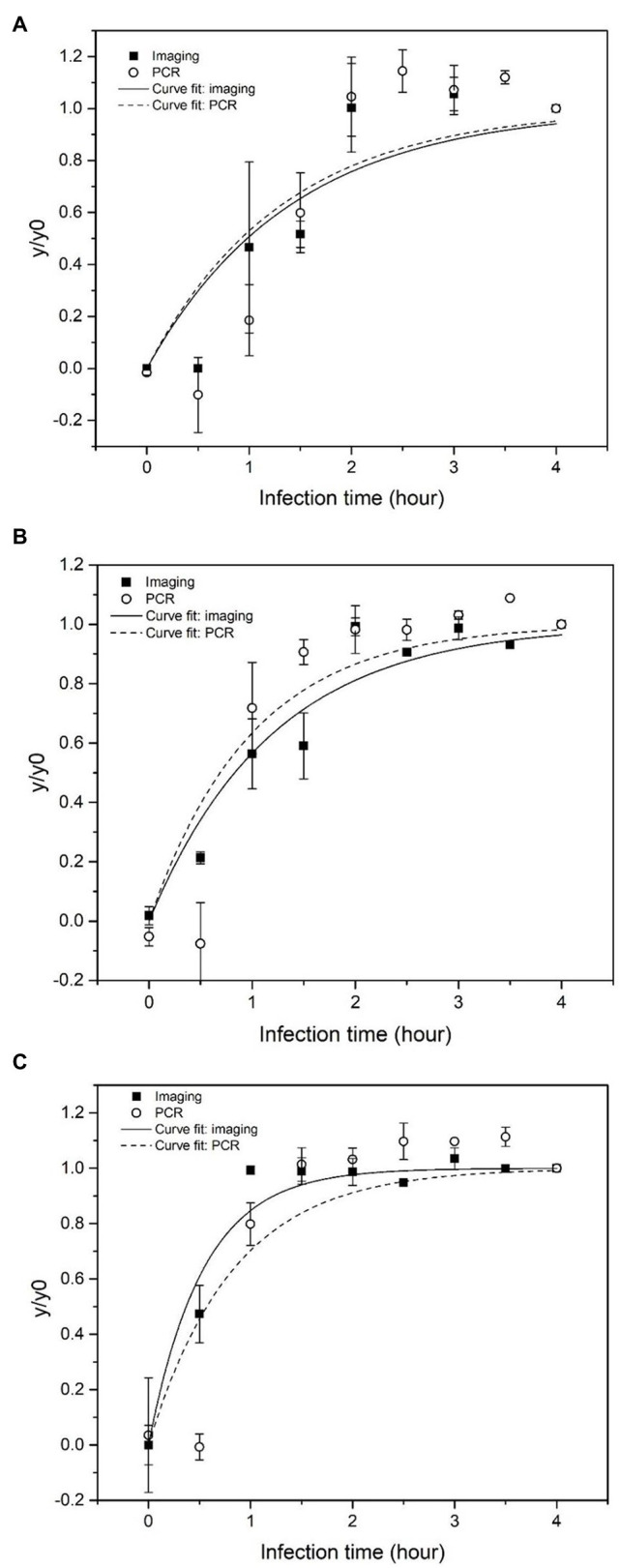
Normalized concentration of phage particles and phage DNA obtained based on imaging (log particle/image) and qPCR (log pg/ml), respectively as a function of infection time. *Escherichia coli* BL21 was enriched for 4 h prior to infection. The initial concentration of *E. coli* was **(A)** 10 CFU/ml, **(B)** 10^2^ CFU/ml, and **(C)** 10^3^ CFU/ml.

For comparing the sensitivity of the imaging and qPCR approaches, amplified phages following infection were detected using both quantitative imaging and qPCR. Amplification of phages has been previously quantified using quantitative-PCR methods (qPCR; Edelman and Barletta, 2003). In addition, detection of bacteria based on measuring phage amplification using qPCR has been reported in previous studies (Stanley et al., 2007; Kutin et al., 2009; Park et al., 2013; Garrido-Maestu et al., 2019; Malagon et al., 2020). To validate and benchmark this novel imaging-based method, bacteriophage concentration at each time point was also quantified by qPCR. The normalized values (*y*/*y*_0_) of phage level quantified by the imaging and PCR, respectively, at each infection time point are shown in Figures 5A–C, when the initial bacteria concentrations were 10–10^3^ CFU/ml, respectively.

The phage amplification rates (*k*) obtained from the imaging and qPCR method were presented in Table 1. With 10 CFU/ml initial *E. coli* concentration, the *k* value from both methods was comparable at around 0.7 s^−1^. When the initial *E. coli* concentration increased to 10^2^ CFU/ml, the *k* value from the imaging method increased to 0.835 s^−1^, while the *k* value from qPCR was 1.112 s^−1^. When the initial *E. coli* concentration was 10^3^ CFU/ml, the *k* value from the imaging method (1.879 s^−1^) was higher than that obtained from qPCR (1.190 s^−1^).

**Table 1:** Amplification rates of phage particles and phage DNA measured based on the imaging and qPCR measurements, respectively.

Initial bacteria concentration		*k* (s^−1^)	$\frac{k\;i}{k\;p}$	SSE	*R*-squared	Adj *R*-sqr	RMSE
10 CFU/ml	Imaging	0.707	0.938	0.204	0.839	0.839	0.184
	qPCR	0.754		0.527	0.754	0.754	0.257
10^2^ CFU/ml	Imaging	0.835	0.751	0.072	0.934	0.934	0.095
	qPCR	1.112		0.129	0.910	0.910	0.127
10^3^ CFU/ml	Imaging	1.878	1.578	0.046	0.955	0.955	0.075
	qPCR	1.190		0.306	0.802	0.802	0.209

The ratio of amplification rates from imaging (*k*_i_) and qPCR (*k*_p_) were calculated for the initial bacterial concentrations of 10–10^3^ CFU/ml. k, rate of bacteriophage amplification; *k*_i_/*k*_p_, ratio of amplification rates from imaging (*k*_i_) and qPCR (*k*_p_); SSE, sum of squares error; Adj R-sqr, adjusted R-squared; and RMSE, root mean square error.

The curve fitting results (Figure 5) show that phage quantification by imaging and qPCR approaches follows a similar trend. The amplification rate (*k*) from imaging and qPCR methods increased with an increase in the concentration of initial inoculum of bacteria, which suggested that relatively higher titer of bacterial host speeded up the amplification of the phages. The qPCR method gave a slightly higher *k* value when bacteria concentration was 10 and 10^2^ CFU/ml, with the ratio of *k*_i_ to *k*_p_ equal to 0.938 and 0.751, respectively. For the bacteria concentration of 10^3^ CFU/ml, the amplification rate detected by imaging was significantly higher with the *k*_i_/*k*_p_ of 1.578. With the initial *E. coli* concentration of 10 CFU/ml, the increase in phage level was detected after 1 h of infection using both imaging and PCR methods. However, the increase in phage level quantified by imaging was observed after the first 30 min of the infection with the initial bacterial concentration of 10^2^ and 10^3^ CFU/ml. In the case of qPCR, the increase in phage DNA level was detected after 1 h of infection.

### Quantification of Amplified Phages Using Imaging and qPCR

Plaque assay is the gold standard for the determination of phage titer. The method involves phage infection on immobilized target bacteria in a molten soft agar overlayed on the nutrient agar. After several hours of incubation, progeny phages generated from an initial infection will infect and lyse nearby bacteria and create a clearance zone called a “plaque” on the opaque lawn of inoculated bacteria. The plaque assay provides an assessment of viable plaque-forming units to represent the concentration of viable phages in a solution, and therefore, it is considered the gold standard for the determination of bacteriophage titer (Anderson et al., 2011).

**Figure 6:**
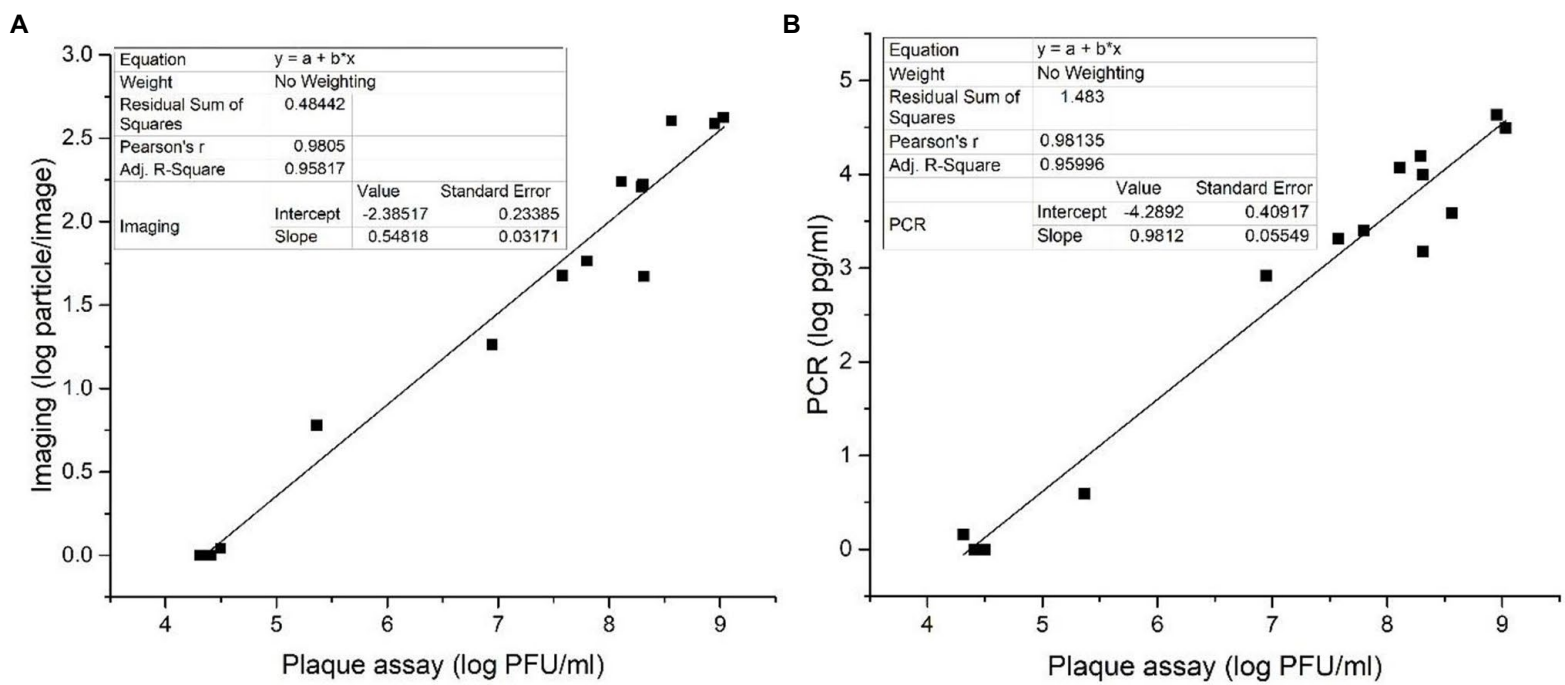
Correlations between **(A)** bacteriophage particle numbers obtained based on imaging measurements and **(B)** bacteriophage DNA concentrations obtained based on qPCR measurements with the phage plaque count measured based on the standard plaque assay.

The data sets obtained with PCR and imaging assays were correlated with the plaque assay. Figure 6 depicts the correlation between the imaging method (Figure 6A) and qPCR (Figure 6B) with the plaque assay. The linear relationship of log phage particle/image against log PFU/ml was *Y* = 0.548*X*−2.385, *R*^2^ = 0.958 (Figure 6A). The linear relationship of log DNA concentration in pg./ml against log PFU/ml was *Y* = 0.981*X*−4.289, *R*^2^ = 0.959 (Figure 6B). In both cases, *R*^2^ values were around 0.96, illustrating a good linear correlation between both the imaging and qPCR methods with the standard plaque assay. Even though plague assay is considered the gold standard for phage enumeration, it requires extended incubation time (between 16 and 20 h). Therefore, more rapid detection methods like qPCR and imaging methods are preferable in foodborne pathogen detection applications.

In addition to detection of amplified phages following infection of target bacteria using imaging and RT-PCR, there have been efforts made to detect phages in water (Rajnovic et al., 2019; Charm Sciences Inc, 2022). These methods have been motivated by the use of coliphage as an indicator of fecal contamination. Since the objective of these methods is to detect phages, these methods typically require an addition of large concentration of host bacteria to the phage sample. Thus, analytically these approaches are designed to enable detection of low titer of phages using high concentration of host bacteria, while the method developed in this study is aimed at detection of low concentration of target bacteria using phages. The method to detect phages have also used optical methods such as changes in optical density based on interactions of phages with high titer of host bacteria. However, these methods have only been tested with samples without the presence of food particles or other interferences that may limit the use of these optical approaches.

Overall, the developed bacterial detection method based on phage amplification and fluorescence imaging provide rapid and sensitive results as it enables the detection of *E. coli* at 10 CFU/ml within 6 h.

### Detection of Bacteria in Food Samples

**Figure 7:**
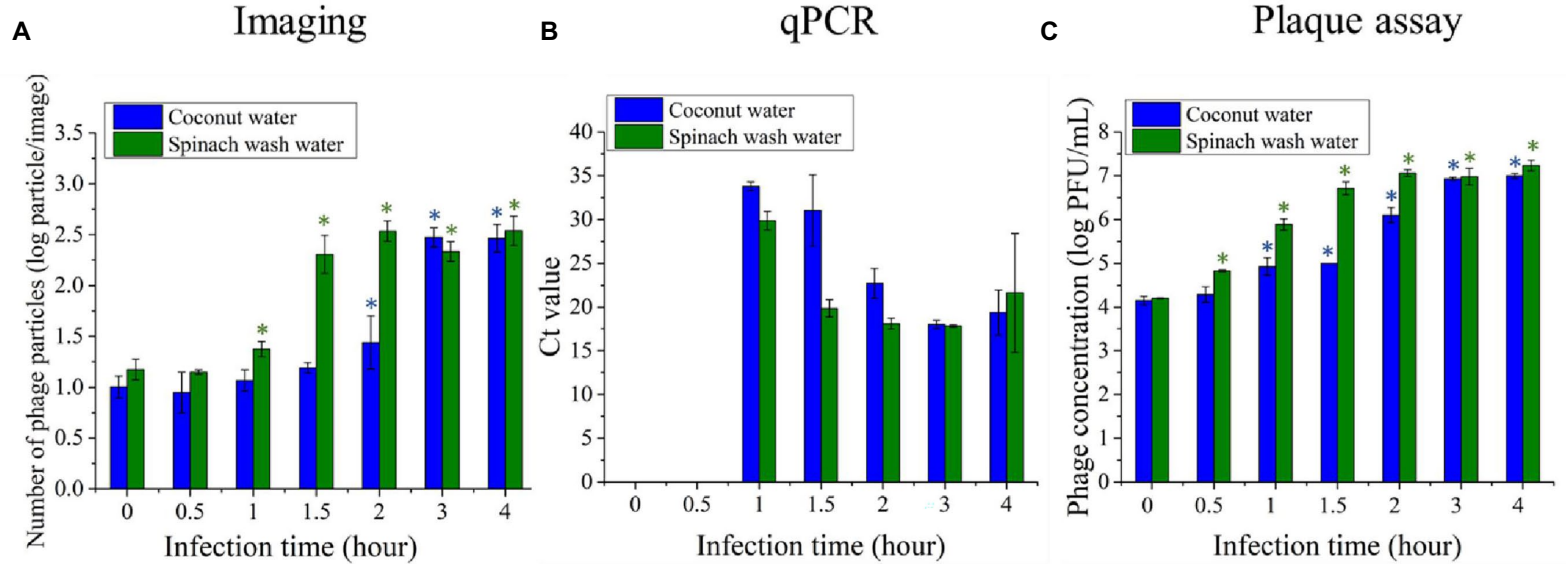
Quantifications of T7 phages amplification after 0 min, 30 min, 1 h, 1 h 30 min, 2 h, 3 h, and 4 h infection of 10 CFU/ml concentration of *E. coli* BL21 cells with T7 phages at a concentration level of 10^4^ PFU/ml. *Escherichia coli* BL21 cells were enriched for 4 h prior to infection with T7 phage. The phage amplification was measured based on **(A)** imaging, **(B)** qPCR, and **(C)** plaque assay. Treatments with “*” are significantly different (*p* < 0.05) from control at 0 min infection. Error bars indicate ±SD of means.

Two model food systems, i.e., coconut water and simulated spinach wash water, were selected to evaluate the effectiveness of the phage amplification-based detection of contaminating bacteria in food samples. These samples were selected based on the unmet needs in the beverage and fresh produce industries to provide rapid detection of bacteria contaminants and our prior experience with these model systems (Tilton et al., 2019; Yang et al., 2020). A 10 CFU/ml of *E. coli* BL21 was inoculated in two selected food samples. After initial enrichment and infection with T7 phages, the amplified phages were detected using the imaging, qPCR, and the standard plaque assay. Figure 7A shows the average number of phage particles per image following amplification of the phages after incubation with the *E. coli* contaminated coconut and spinach samples. The plot shows changes in the number of phage particles after 0, 30 min, 1 h, 1 h 30 min, and 2–4 h of infection. At the infection time of 0 min, representing the initial T7 inoculum, around 10 and 15 particles were observed on the images/field of coconut and spinach wash water samples, respectively. The particles observed at the initial inoculation time might result from some fluorescent residues from the coconut water and spinach wash water that are auto-fluorescent or can be stained by SYBR green. For coconut water, 2 h of infection was required to significantly increase (*p* < 0.05) in the number of phage particles from the initial background level. The particles increase to around 300 with an infection time of 3 h. For the spinach wash water samples, there was a significant increase (*p* < 0.05) in the number of phage particles within 1 h of infection, and the phage particles increased to around 300 particles after 2 h. No significant increase in the phage particles per image was observed with an extended incubation time greater than 2 h for the spinach wash water samples. For both coconut water and spinach wash water samples without *E. coli* inoculation, there was no significant increase in the number of phage particles after 4 h of infection. These results demonstrate that the imaging method can detect amplification of phages in coconut water and spinach wash water, and 10 CFU/ml of *E. coli* in coconut water and spinach wash water can be detected with an infection period of 2 and 1 h, respectively, using the imaging approach.

A qPCR was also performed to detect the increase in concentration of T7 phage DNA, which represents phage amplification. The level of phage DNA was expressed in terms of the cycle threshold value (Ct value), which is defined as the number of reaction cycles required for the fluorescence signal to exceed the threshold. The samples with lower Ct values contain a higher T7 phage concentration. Figure 7B shows the average Ct values for coconut water and spinach wash water samples with *E. coli* inoculation. The phage DNA level in the samples with the infection time of 0 and 30 min were not detectable as the fluorescence signals were lower than the threshold. With a 1-h infection, the average Ct value of the coconut sample was 33.79, while the average Ct value of the spinach samples was 29.88. After 1 h of infection, there was an increase in the level of T7 phage DNA indicating T7 phage amplification. Increasing the infection time to 2 h decreased the Ct value of the coconut water and spinach wash water samples to 31 and 19.8, respectively. The lower Ct values indicate the higher level of the target DNA; therefore, the amplification T7 phage rate was relatively higher in the spinach wash water than those of the coconut water.

The concentration of T7 phage in both types of food samples was also quantified using the plaque counting assay (Figure 7C). The significant increase (*p* < 0.05) in T7 phage concentration was observed when the infection time was 30 min and 1 h for spinach wash water and coconut water samples, respectively. The amplification rate of T7 phage was higher in the spinach wash water samples, which is consistent with the results of phage quantification performed using the imaging and qPCR methods.

The results suggest that the imaging-based method has the sensitivity to detect 10 CFU/ml of *E. coli* in the selected food samples, coconut water, and spinach wash water. In complex food samples, the background signal from the food components is considered a constraint for the detection of pathogens since it reduces the detection sensitivity. For the imaging-based detection approach, the centrifugal step removed some of the large debris from food products along with the bacterial debris. Despite this step, small fragments of the food particles did contribute to a small detectable background in the case of samples imaged at an infection time of 0 h. Despite this background, the imaging approach was able to detect 10 CFU/ml within 6–7 h of the total assay time, including enrichment of the inoculated bacterial samples.

The presence of food matrices may either affect the growth of bacteria during enrichment or the infection and amplification of T7 phages. Therefore, total detection times are presented as a function of selected food matrixes for different detection methods in Table 2. The phage amplification rate was lower than using PCR and plaque assay in a coconut water sample for the imaging method. Thus, a longer infection time was required to detect bacteria in coconut water using an imaging approach. It is possible that components of coconut water may slow the growth of *E. coli* which is in contrast with the study of Awua (2012) that fresh coconut water is favorable for the survival and growth of *E. coli* (Awua, 2012). Another possibility is that the components in coconut water decelerate the infection and amplification of the phages. In the case of spinach water samples, the infection time was shortest for the plaque assay (0.5 h), while both the PCR and imaging assay required 1 h of infection time.

**Table 2:** The infection time and the total assay time (rounded to nearest hour) for the detection of 10 CFU/ml of *E. coli* in TSB, coconut water, and spinach wash water.

	Imaging			PCR			Plaque assay		
	TSB	Coconut water	Spinach water	TSB	Coconut water	Spinach water	TSB	Coconut water	Spinach water
Infection time (h)	1	2	1	1	1	1	1	1	0.5
Total time (h)	6	7	6	7	7	7	>16	>16	>16

Even though the imaging method requires a longer infection time to observe phage amplification for the coconut water sample, it saves 1 h of total operation time than those required for the qPCR method. The imaging method requires 7 and 6 h to detect 10 CFU/ml of *E. coli* in coconut water and spinach wash water. The qPCR method requires 7 h to detect the same concentration of *E. coli* in both samples. The longer time required for the qPCR method is a result of the fact that a DNA extraction step was necessary, and a 40-cycle amplification during PCR takes almost 1 h 30 min.

On the other hand, the imaging approach has the necessary sensitivity to detect phage particles with a simple method based on filtration and imaging. Thus, it saves both time and resources required for the extraction of DNA and molecular laboratory environment requirements. Moreover, the protocol of the imaging method is relatively more straightforward and does not require well-trained personnel for the operation, which can make it more cost-efficient. Another key advantage of the imaging-based method is the automation of imaging procedures, such as automated scanning of the slides is now routine in many commercial imaging systems (Conze et al., 2010; Lu et al., 2019). These advantages of the imaging method also enable the possibility to develop a portable detection tool that can be operated in the field.

A possible limitation of the imaging method is that the filtration setup we were using may not be suitable to test multiple samples simultaneously. This constraint may be mitigated by applying a control pump and flow regulators to have multiple channels for simultaneous processing of samples. A high-throughput filtration system has been developed as a screening tool for viruses. This setup is a 96-well plate format, allowing multiple samples to be filtered simultaneously (Tang et al., 2020). The requirement for the fluorescence microscope might be considered another constraint of this method. However, smartphone-based fluorescence microscope developments with high magnification allow low-cost and convenient fluorescence imaging of viral particles (Cho et al., 2016; Müller et al., 2018; Chung et al., 2021). These technologies may further develop this detection method to have a more straightforward setup and be applicable for on-site bacterial pathogen detection.

The success of the image analysis-assisted enumeration of bacteriophage for detecting *E. coli* can lead to rapid, cost-effective, and easy-to-use bacteria detection methods applicable in food and agricultural industries. The detection approach developed in this study does not require complicated sample preparations. Using wild-type bacteriophage, T7 without any genetic modification opens opportunities to develop other phage-based assays using environmentally isolated phages.

## Conclusion

T7 phage particles can be visualized using SYBR Green I nucleic acid stain and fluorescence microscope. The number of the phage particles in the fluorescence images can be enumerated using image processing software, which allows the rapid enumeration of phage amplification upon infection of the target bacteria. The rate of phage amplification detected by the image analysis-assisted enumeration is comparable with the qPCR method. The phage quantification results obtained from the imaging method shows high correlation with the plaque assay method. With this approach, *E. coli* at 10 and 10^2^ CFU/ml in TSB can be detected after 4-h enrichment and 1 h infection, and 10^3^ CFU/ml of *E. coli* can be detected after 4-h enrichment and 30 min infection. The imaging detection method can detect 10 CFU/ml of *E. coli* in selected food matrices, coconut water, and spinach wash water, within 7 and 6 h, respectively. The sensitivity of the imaging approach also eliminates the need for extensive sample preparation and secondary amplification of the target phage DNA using RT-PCR or other amplification methods. This image analysis-based rapid bacteria detection approach can be further developed to be applicable for pathogen detection in agricultural industries and other foodborne pathogens.

## Data Availability Statement

The original contributions presented in the study are included in the article/Supplementary Material, further inquiries can be directed to the corresponding author.

## Author Contributions

NW designed the study and performed the experiments, analyzed the data, and wrote the manuscript. XY designed the study and performed the experiments. NN and GY conceived, designed, and supervised the study. All authors contributed to the article and approved the submitted version.

## Funding

This study was supported by USDA National Institute of Food and Agriculture (USDA-NIFA) grant No. 2015-68003-23411.

## Conflict of Interest

The authors declare that the research was conducted in the absence of any commercial or financial relationships that could be construed as a potential conflict of interest.

## Publisher’s Note

All claims expressed in this article are solely those of the authors and do not necessarily represent those of their affiliated organizations, or those of the publisher, the editors and the reviewers. Any product that may be evaluated in this article, or claim that may be made by its manufacturer, is not guaranteed or endorsed by the publisher.
