# Supplementary material for: Quantitative Imaging of Bacteriophage Amplification for Rapid Detection of Bacteria in Model Foods
Source: Front Microbiol. 2022 Mar 4;13:853048. doi: 10.3389/fmicb.2022.853048 (PMC8931685; doi:10.3389/fmicb.2022.853048)
Supplement: Supplementary file 1 [file Table_1.DOCX]

Supplementary Material

# Customized MATLAB code for phage particles enumeration from fluorescence images

filedir = 'xxx’; % File path of the folder containing fluorescence images in JPEG format

list_filename = dir(fullfile(filedir,'*.jpg'));

list_numObjects = cell(2,size(list_filename,1));

for i=1:size(list_filename,1)

A_jpg=imread(list_filename(i).name);

se1 = strel('disk',30); %creates a disk-shaped flat structuring element with specified neighborhood of 30 pixels

A2 = imtophat(A_jpg,se1); % performs morphological top-hat filtering on the grayscale or binary image

B = medfilt2(A2,[3,3]); %reduce noise from background

C = adapthisteq(B); %improve the visibility level of foggy image

D = wiener2(C,[3,3]); % remove fine noise

T = adaptthresh(D,0.4,'ForegroundPolarity','bright'); %compute adaptive threshold and display the local threshold image with the sensitivity of 0.4 with the foreground brighter than the background.

BW = imbinarize(B,T); %Binarize 2-D grayscale image by thresholding

BW_unit8 = im2uint8(BW); %Convert image to 8-bit unsigned integers

[labeled,numObjects] = bwlabel(BW,4);

list_numObjects{1,i} = list_filename(i).name;

list_numObjects{2,i} = numObjects;

end

**2 The examples of images obtained from the initial *E. coli* concentration of 10 and 10^3^ CFU/mL**

**
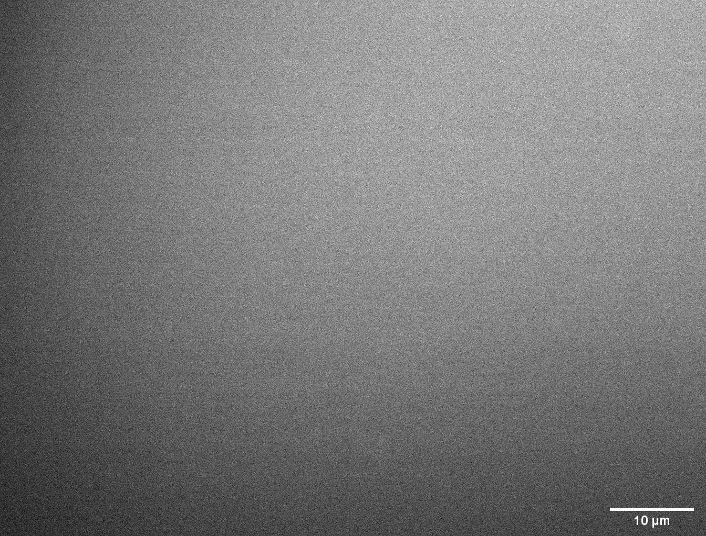

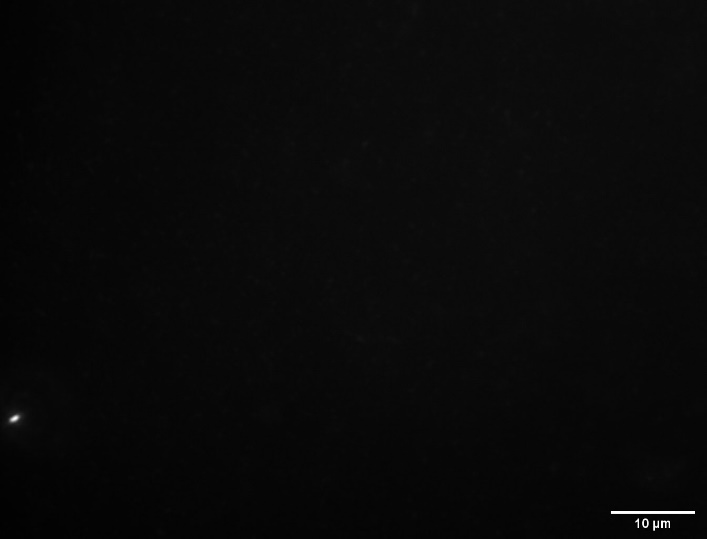
**

30 m

0 m

**
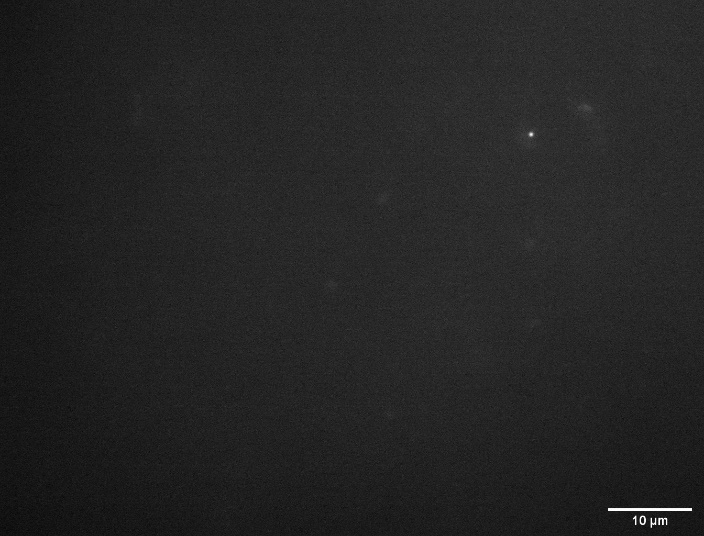

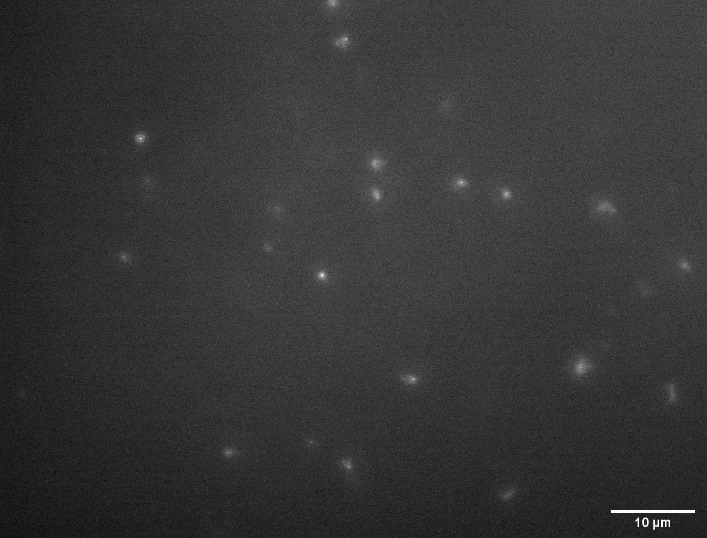
**

2h

1h

**
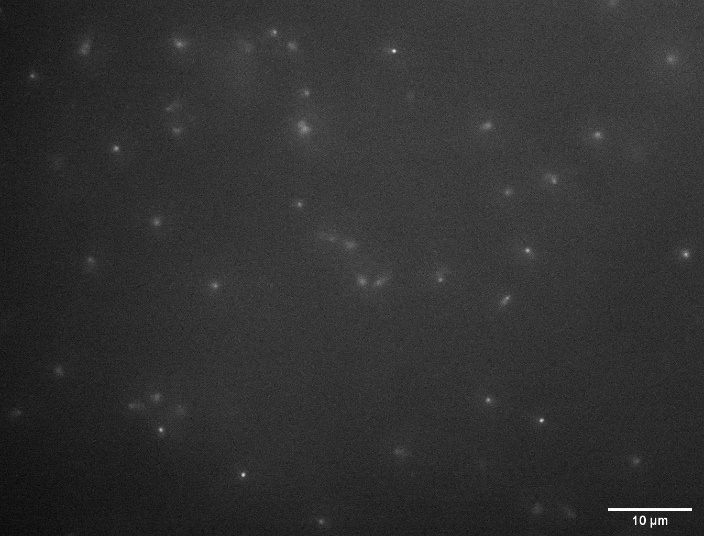

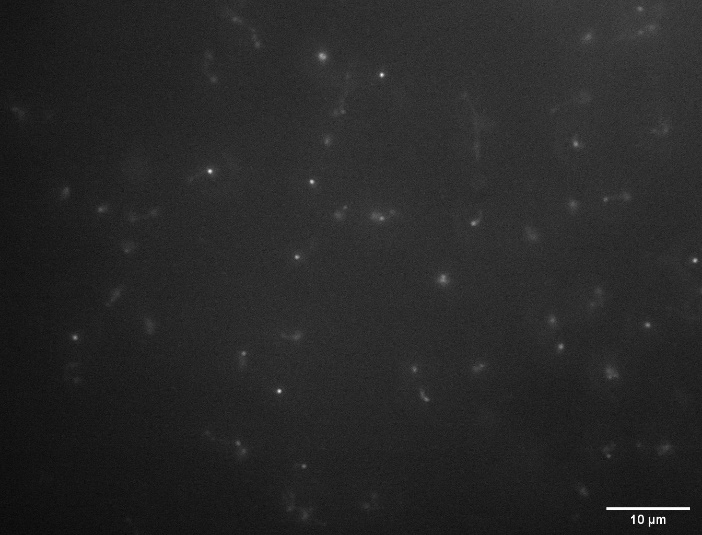
**

4h

3h

**Figure S1** Fluorescence images of the bacteriophage particles after 0 minutes, 30 minutes, 1 hour, 2 hours, 3 hours, and 4 hours of infection of *E. coli* BL21 (10 CFU/ml initial concentration) with 10^4^ PFU/ml of T7 phages. *E. coli* cells were enriched for 4 hours prior to infection with phages.

**
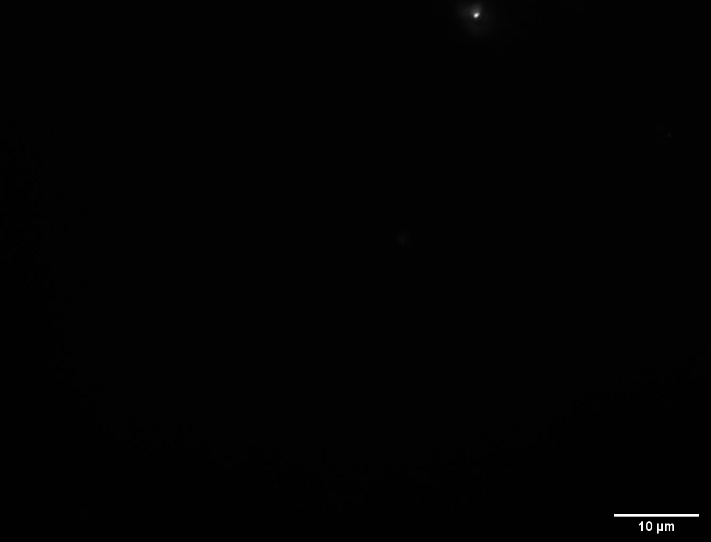

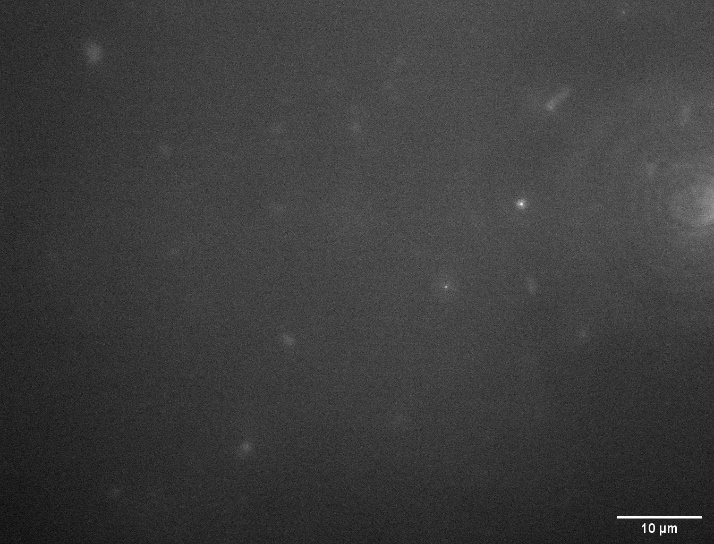
**

30 m

0 m

**
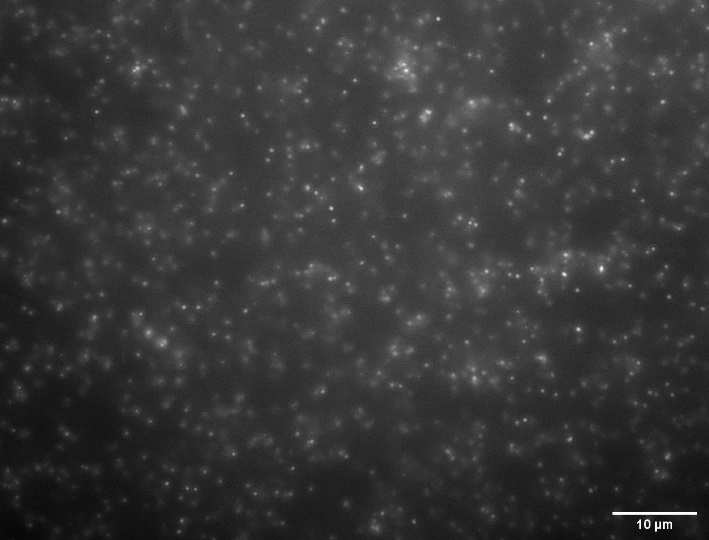

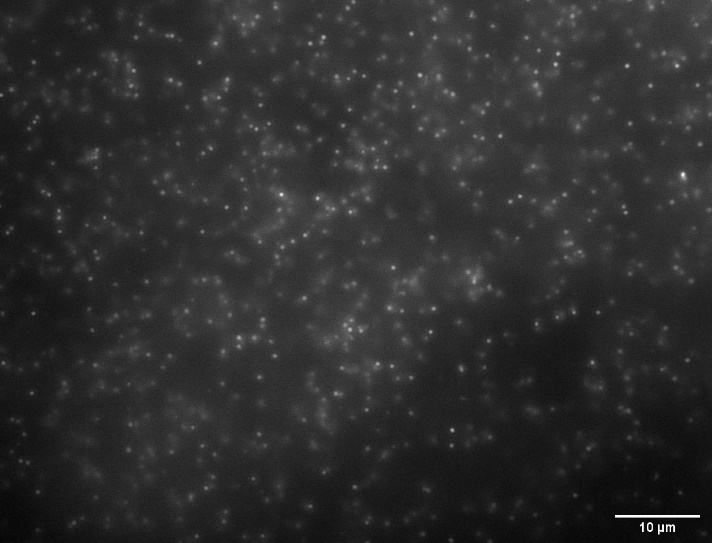
**

2 h

1 h

**
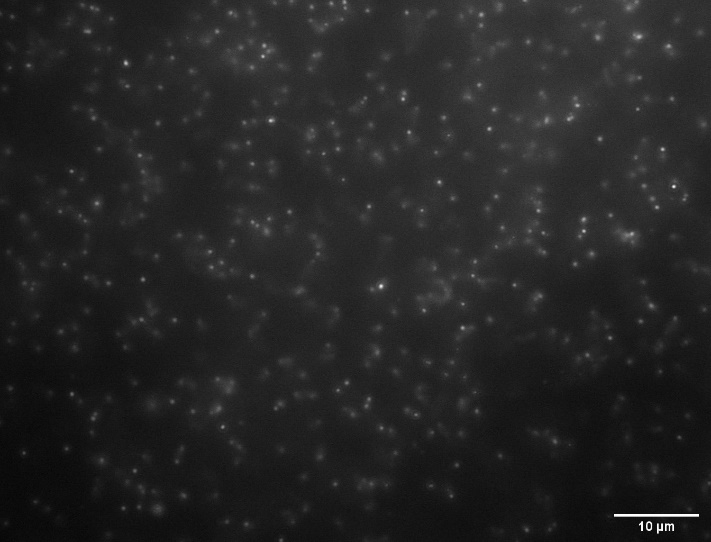

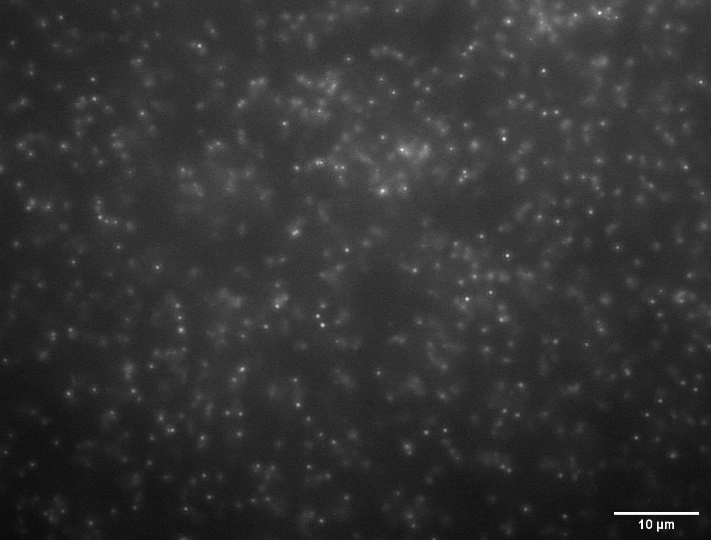
**

4 h

3 h

**Figure S2** Fluorescence images of the bacteriophage particles after 0 minutes, 30 minutes, 1 hour, 2 hours, 3 hours, and 4 hours of infection of *E. coli* BL21 (10^3^ CFU/ml initial concentration) with 10^4^ PFU/ml of T7 phages. *E. coli* cells were enriched for 4 hours prior to infection with phages.
